# Robust interaction of IFT70 with IFT52–IFT88 in the IFT-B complex is required for ciliogenesis

**DOI:** 10.1242/bio.033241

**Published:** 2018-04-13

**Authors:** Ryota Takei, Yohei Katoh, Kazuhisa Nakayama

**Affiliations:** Department of Physiological Chemistry, Graduate School of Pharmaceutical Sciences, Kyoto University, Sakyo-ku, Kyoto 606-8501, Japan

**Keywords:** Cilia, IFT-B complex, IFT70, IFT22

## Abstract

In the intraflagellar transport (IFT) machinery, the IFT-B and IFT-A complexes mediate anterograde and retrograde ciliary protein trafficking, respectively. Among the 16 subunits of the IFT-B complex, several subunits are essential for ciliogenesis, whereas others, which are associated peripherally with the complex, are dispensable for ciliogenesis but play a role in protein trafficking. *IFT22*-knockout (KO) cells established in this study demonstrated no defects in ciliogenesis or ciliary protein trafficking. In stark contrast, *IFT70A* and *IFT70B* double-knockout cells did not form cilia, even though IFT70 is associated peripherally with the IFT-B complex via the IFT52–IFT88 dimer, and other IFT-B subunits assembled at the ciliary base in the absence of IFT70. Exogenous expression of either IFT70A or IFT70B restored the ciliogenesis defect of *IFT70*-KO cells, indicating their redundant roles. IFT70 has 15 consecutive tetratricopeptide repeats (TPRs) followed by a short helix (α36). Deletion of the first TPR or α36 of IFT70A greatly reduced its ability to interact with the IFT52–IFT88 dimer. Exogenous expression of any of the IFT70A deletion mutants in *IFT70*-KO cells could not restore ciliogenesis. These results show that IFT70 plays an essential role in ciliogenesis, although it is dispensable for assembly of the residual IFT-B subunits.

## Abstract (precis)

**Summary:** IFT70 is a subunit of the IFT-B complex involved in anterograde trafficking of ciliary proteins, including tubulins. *IFT70*-knockout cells failed to form cilia, indicating its essential role in tubulin trafficking.

## INTRODUCTION

Cilia are appendage organelles that protrude from the surfaces of a variety of eukaryotic cells, and are maintained by the presence of the microtubule-based axoneme. Specific proteins exist within cilia and on the ciliary membrane. The cilioplasm and ciliary membrane are separated from the cytoplasm and plasma membrane by the transition zone, which serves as a diffusion/permeability barrier at the ciliary base (Ishikawa and Marshall, 2011; Jensen and Leroux, 2017). The assembly of cilia as well as signal transduction from cilia, such as Hedgehog (Hh) signaling, is supported by the intraflagellar transport (IFT) machinery, which is composed of the IFT-A and IFT-B complexes and the BBSome. Owing to the crucial roles played by cilia in various physiological and developmental processes, defects in cilia result in the ciliopathies, such as Bardet-Biedl syndrome (BBS), which display a broad range of symptoms (Braun and Hildebrandt, 2017; Brown and Witman, 2014).

**Fig. 1.**
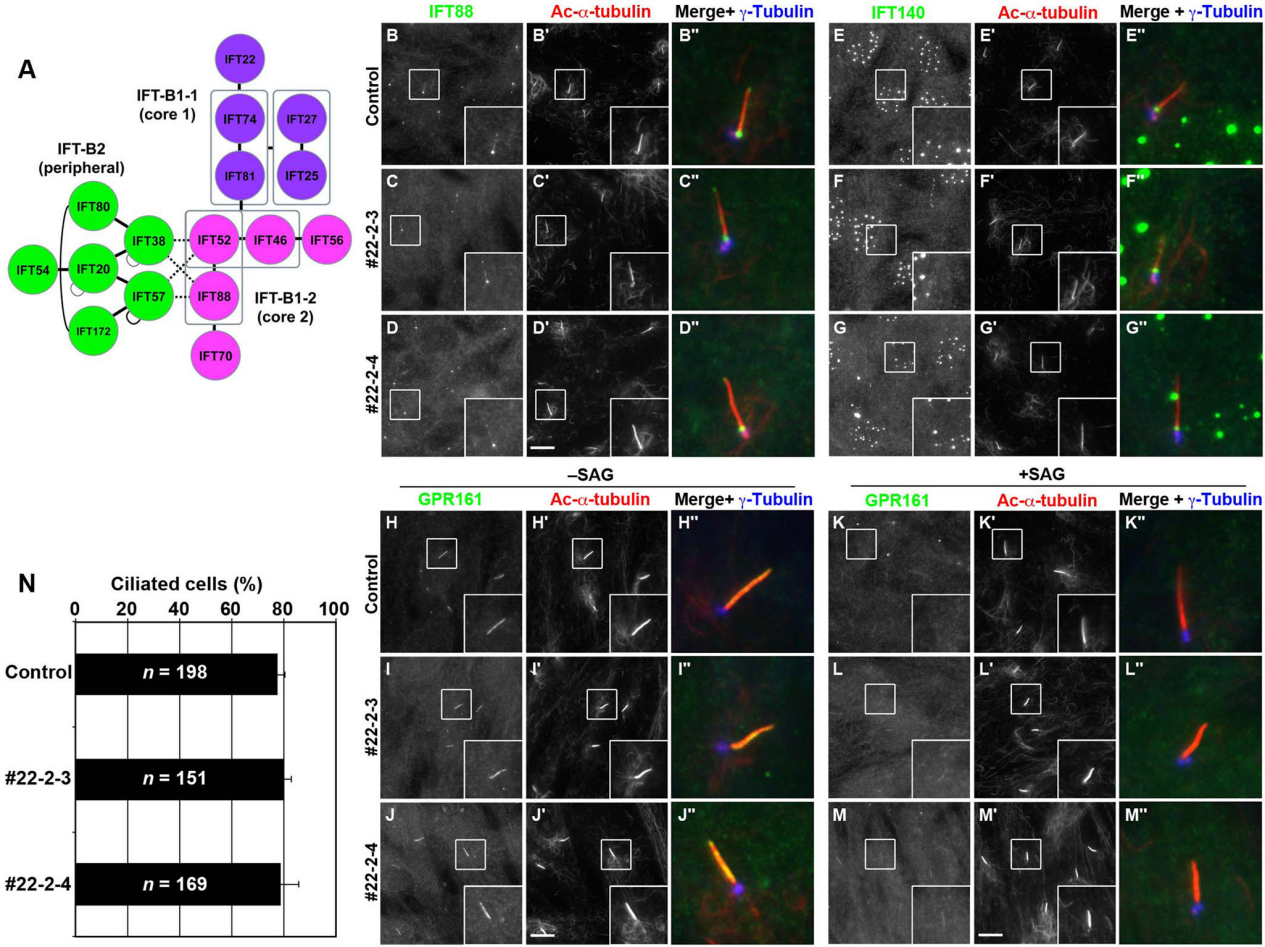
***IFT22*-KO cell lines show no apparent defects.** (A) Schematic representation of the architecture of the IFT-B complex predicted from our previous study. (B–M) Control RPE1 cells (B,E,H,K) and the *IFT22*-KO cell lines #22-2-3 (C,F,I,L) and #22-2-4 (D,G,J,M) were serum-starved for 24 h. (B–G) The cells were then directly triple immunostained for either IFT88 (B–D) or IFT140 (E–G), together with Ac-α-tubulin (B′–G′), and γ-tubulin (B″–G″). (H–M) The serum-starved cells were incubated for a further 24 h in the absence (–SAG) or presence (+SAG) of 200 nM SAG, and triple immunostained for GPR161 (H–M), Ac-α-tubulin (H′–M′), and γ-tubulin (H″–M″). Scale bars: 10 µm. Merged, enlarged images of the boxed regions are shown in (B″–M″). (N) Ciliated cells of control RPE1 cells and the *IFT22*-KO cell lines #22-2-3 and #22-2-4 were counted, and percentages of ciliated cells are represented as bar graphs. Values are shown as means±s.e. of three independent experiments. In each set of experiments, 47–90 cells for each sample were analyzed, and the total number of cells analyzed (*n*) for each sample is shown.

The IFT-A complex is composed of six subunits and mediates retrograde ciliary protein trafficking driven by the dynein-2 motor protein complex, whereas the IFT-B complex composed of 16 subunits, mediates anterograde trafficking driven by the kinesin-2 motor (Nakayama and Katoh, 2018; Prevo et al., 2017; Taschner and Lorentzen, 2016). Recently, we as well as others independently delineated the architecture of the IFT-B complex (Boldt et al., 2016; Katoh et al., 2016; Taschner et al., 2016). The entire IFT-B complex can be divided into two subcomplexes; the IFT-B2 (peripheral) and IFT-B1 (core) subcomplexes composed of six and ten subunits, respectively (see Fig. 1A).

Among the IFT-B subunits, knockout (KO) of particular subunits, such as IFT20, IFT38, and IFT88, results in the ‘no cilia’ phenotype (Katoh et al., 2017, 2016), which probably results from a lack of tubulin trafficking in the absence of the functional IFT-B complex (Lechtreck, 2015). On the other hand, KO of other IFT-B subunits, such as IFT25, IFT27, and IFT56, which are located at the periphery of the IFT-B1 subcomplex (see Fig. 1A), has a marginal effect on ciliogenesis per se, but impairs trafficking of specific ciliary proteins (Eguether et al., 2014; Funabashi et al., 2017; Keady et al., 2012) [for review, see Nakayama and Katoh, (2018)].

Although IFT22 and IFT70 are also located at the periphery of the IFT-B1 subcomplex via the IFT74–IFT81 dimer and the IFT52–IFT88 dimer, respectively (see Fig. 1A), little is known about their roles, particularly in vertebrate cilia. In this study, we therefore established *IFT22*-KO and *IFT70*-KO cell lines from human telomerase reverse transcriptase-immortalized retinal pigment epithelial 1 (hTERT-RPE1) cells. KO of IFT22 led to no discernible phenotypic changes, whereas rather unexpectedly, KO of IFT70 resulted in the ‘no cilia’ phenotype. We therefore investigated the interactions of IFT70 with other subunits in detail, and found that the entire region of IFT70 is essential for its interaction with the IFT52–IFT88 dimer of the IFT-B complex and that robust interaction of IFT70 with IFT52–IFT88 is essential for ciliogenesis.

## RESULTS

### IFT22-KO cells have no discernible phenotype

IFT27 (also known as RABL4 and BBS19) and IFT22 (also known as RABL5), which are components of the IFT-B complex, are small GTPases, and therefore might serve as molecular switches in ciliary protein trafficking. IFT27/RABL4 forms a dimer with IFT25 to interact with the IFT74–IFT81 dimer (Fig. 1A) and was shown to participate in retrograde ciliary protein trafficking via connecting the IFT machinery to the BBSome (Eguether et al., 2014; Huet et al., 2014; Liew et al., 2014). We as well as others recently showed that RABL2, which is not an integral component of the IFT-B complex, localizes to the ciliary base and regulates ciliary assembly via interacting with the IFT74–IFT81 dimer (Kanie et al., 2017; Nishijima et al., 2017). IFT22/RABL5 also interacts with IFT74–IFT81 (Fig. 1A) (Katoh et al., 2016). In *Caenorhabditis elegans*, disruption of the *IFTA-2*/*IFT22* gene did not result in overt abnormalities of ciliary assembly or IFT trafficking (Schafer et al., 2006). In contrast, RNAi-knockdown of RABL5/IFT22 in *Trypanosoma brucei* and in *Chlamydomonas reinhardtii* was reported to result in short flagella with increased expression levels of flagellar IFT proteins (Adhiambo et al., 2009; Silva et al., 2012). However, there have been no reports on the function of IFT22 in vertebrates. Therefore, here we established *IFT22*-KO cell lines by applying the CRISPR/Cas9 system to hTERT-RPE1 cells (for genotypic characterization of these cell lines, see Fig. S1) and analyzed their phenotype.

Control RPE1 cells and the *IFT22*-KO cell lines #22-2-3 and #22-2-4 were serum-starved to induce ciliogenesis, and then double immunostained for either IFT88 (an IFT-B subunit) or IFT140 (an IFT-A subunit) and acetylated α-tubulin (Ac-α-tubulin), a marker for the ciliary axoneme. The frequency of cilia formation as well as the ciliary length depicted by staining for Ac-α-tubulin was not apparently different between the control RPE1 (Fig. 1B′) and *IFT22*-KO cell lines (Fig. 1C′,D′) (also see Fig. 1N). Neither the localization of the IFT-B (Fig. 1B–D) nor that of the IFT-A protein (Fig. 1E–G) was different between the control and *IFT22*-KO cells. As described previously (Hirano et al., 2017), IFT88 was localized mainly around the base of cilia and faintly along cilia, and IFT140 was mainly found around the ciliary base; note that, as described previously (Hirano et al., 2017; Takahara et al., 2018), the commercially available polyclonal antibody against IFT140 also stained undetermined structures in the nucleus of RPE1 cells; see the manufacturer's website (http://www.ptglab.com/Products/IFT140-Antibody-17460-1-AP.htm).

We then compared the localization of GPR161 between control and *IFT22*-KO cells under basal conditions and under conditions in which Hh signaling was activated. GPR161 is a seven-transmembrane G-protein-coupled receptor that negatively regulates Hh signaling (Mukhopadhyay and Rohatgi, 2014). Under basal conditions, in which Hh signaling is in the ‘off’ state, GPR161 was localized on the ciliary membrane (Fig. 1H). When control cells were treated with Smoothened Agonist (SAG) to stimulate Hh signaling, GPR161 exited cilia (Fig. 1K), resulting in cancelation of the negative regulation (Mukhopadhyay and Rohatgi, 2014). In the *IFT22*-KO cell lines, GPR161 was localized on the ciliary membrane under basal conditions (Fig. 1I,J), and exited cilia under SAG-stimulated conditions (Fig. 1L,M). Thus, we were unable to detect any abnormalities in ciliogenesis or in ciliary protein localization and trafficking in the *IFT22*-KO cell lines, in line with the observations in the *C. elegans IFTA-2* mutant (Schafer et al., 2006).

### ‘No cilia’ phenotype in IFT70-KO cells

An early study by Ou et al. on the neuronal cilia of *C. elegans* suggested that DYF-1 (an IFT70 ortholog) may regulate homodimeric OSM-3 kinesin (an ortholog of KIF17), and showed that *dyf-1* mutants lack the distal, singlet axoneme segment of neuronal cilia, in which homodimeric OSM-3 serves as a motor (Ou et al., 2005). However, we recently showed that human KIF17 interacts with the IFT-B complex via the IFT46–IFT56 dimer, but not via IFT70, and that *KIF17*-KO cells and *IFT56*-KO cells demonstrate no apparent ciliogenesis defects (Funabashi et al., 2017). On the other hand, IFT70-knockdown *Chlamydomonas* and the *fleer* mutant of zebrafish (Fleer is an IFT70 ortholog) were reported to have short cilia/flagella (Fan et al., 2010; Pathak et al., 2007). However, there have been no knockdown or KO studies on mammalian IFT70. The reason for this may be rather simple. Because humans have two IFT70 paralogs, IFT70A and IFT70B (also known as TTC30A and TTC30B, respectively) with approximately 95% amino acid identity, and mice have three paralogs, IFT70A1, IFT70A2, and IFT70B (Howard et al., 2013), knockdown/knockout of a single IFT70 paralog might result in no phenotype change.

**Fig. 2.**
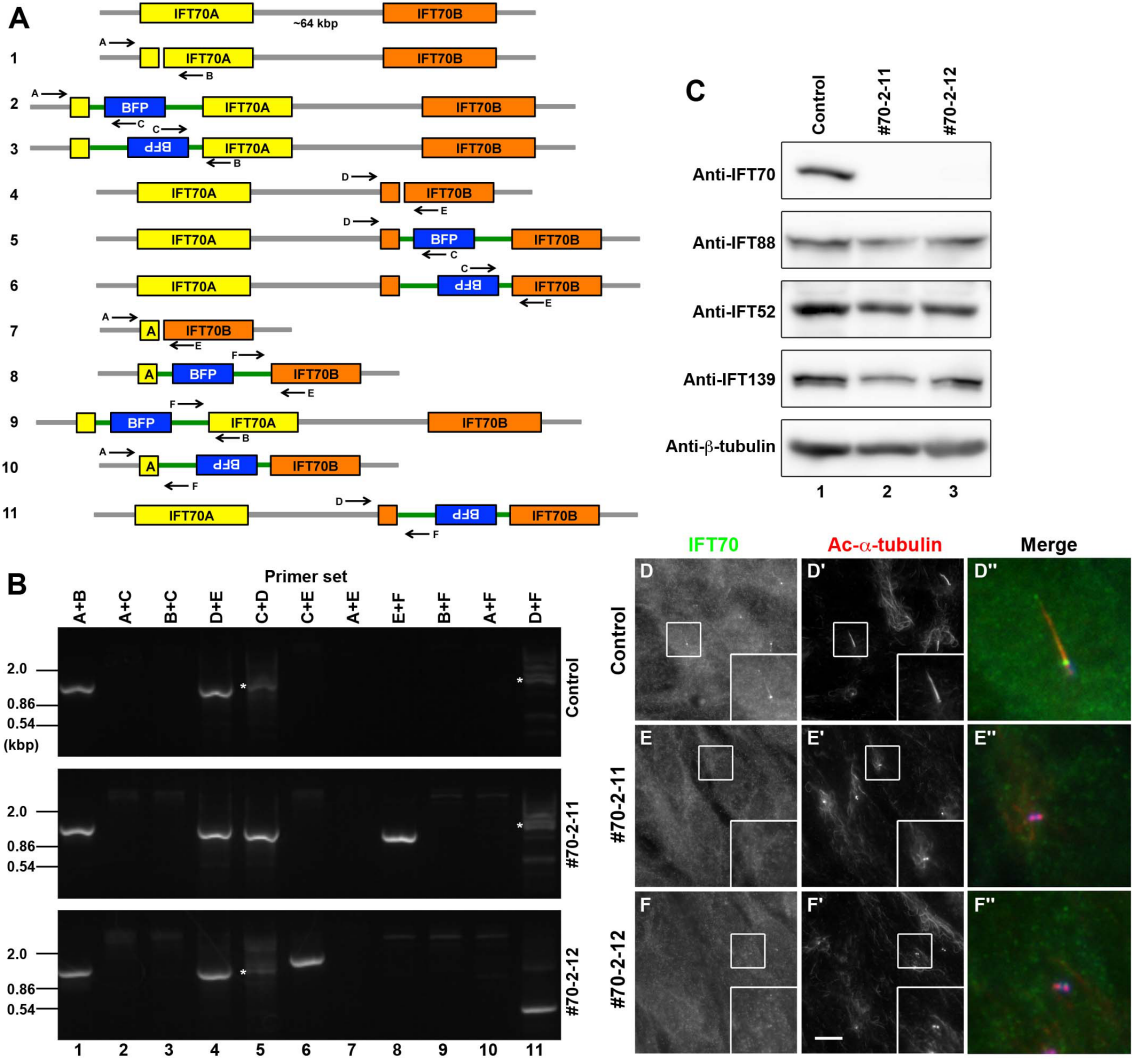
**Characterization of *IFT70*-KO cell lines.** (A) Schematic representation of the possible modes of disruption of the *IFT70A* and *IFT70B* genes. Primer sets used to distinguish among the possible modes of disruption are also shown. Primer sequences are listed in Table S3. (B) Genomic DNA was extracted from control RPE1 cells and from the candidate *IFT70*-KO cell lines #70-2-11 and #70-2-12, and subjected to PCR using the primer sets shown in Fig. 2A (also see Table S3). White asterisks indicate nonspecifically amplified DNA bands. (C) Lysates were prepared from control RPE1 cells and the #70-2-11 and #70-2-12 cell lines, and subjected to immunoblotting analysis using antibodies against IFT70, IFT88, IFT52, IFT139, or β-tubulin. (D–F) Control RPE1 cells (D), and the #70-2-11 (E) and #70-2-12 (F) cell lines were serum-starved to induce ciliogenesis and double immunostained for IFT70 (D–F) and Ac-α-tubulin (D′–F′). Scale bar: 10 µm. Merged, enlarged images of the boxed regions are shown in (D″–F″).

On the other hand, we recently developed a practical strategy of CRISPR/Cas9-mediated targeted gene disruption, which is applicable to hTERT-RPE1 cells and results in a relatively high efficiency of gene disruption (Katoh et al., 2017). In this gene disruption strategy, we adopted a homology-independent knock-in system, rather than homologous recombination (Katoh et al., 2017). We expected that simultaneous KO of the two *IFT70* genes is possible with the same target nucleotide sequence for the *IFT70A* and *IFT70B* genes (see Table S3) by our CRISPR/Cas9 strategy for the following reasons: (i) The human *IFT70A* and *IFT70B* genes have 96% similarity in their nucleotide sequences, at least in the coding region; (ii) both the *IFT70A* and *IFT70B* genes consist of single exons; and (iii) the two loci are only separated from each other by approximately 64 kbp (see Fig. 2A, top row).

Among the candidate *IFT70*-KO lines of hTERT-RPE1 cells, we chose the cell lines #70-2-11 and #70-2-12 for the following analyses. Genomic PCR analysis using various primer sets (Fig. 2A; Table S3) suggested that both #70-2-11 and #70-2-12 have small indels in both the *IFT70A* and *IFT70B* genes (indicated by primer sets A+B and D+E, respectively), in one of the two alleles (Fig. 2B, lanes 1 and 4). In addition, #70-2-11 and #70-2-12 had forward and reverse integration, respectively, of the donor knock-in vector in the other *IFT70B* allele (indicated by primer sets C+D and C+E, respectively) (Fig. 2B, lanes 5 and 6). Direct sequencing of the PCR products confirmed that the #70-2-11 cell line has a ten-nucleotide deletion in one of the *IFT70A* alleles, a one-nucleotide insertion in one of the *IFT70B* alleles, and a forward integration of the donor vector in the other *IFT70B* allele (Fig. S2A); and #70-2-12 has a one-nucleotide deletion in one of the *IFT70A* alleles, a one-nucleotide deletion in one of the *IFT70B* alleles, and a reverse integration of the donor vector in the other *IFT70B* allele (Fig. S2B). Thus, for both the #70-2-11 and #70-2-12 cell lines, among a total of four *IFT70* alleles, we confirmed the disruption of three alleles.

**Fig. 3.**
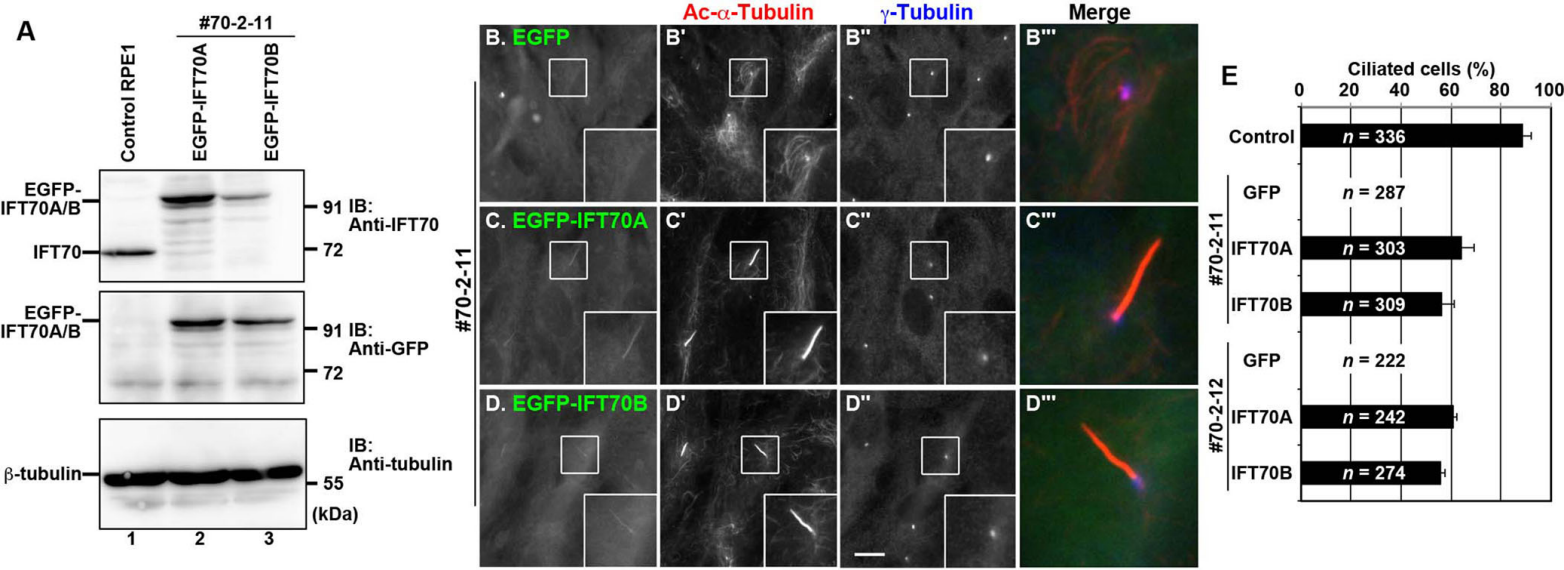
**IFT70A and IFT70B are redundant with respect to ciliogenesis.** (A) Lysates were prepared from control RPE1 cells and the #70-2-11 cell line stably expressing EGFP-IFT70A or IFT70B subjected to immunoblotting analysis using antibodies against IFT70, GFP, or β-tubulin. (B–D) The #70-2-11 cell line stably expressing EGFP (B), EGFP-IFT70A (C), or EGFP-IFT70B (D) were serum-starved to induce ciliogenesis and double immunostained using antibodies against Ac-α-tubulin (B′–D′) and γ-tubulin (B″–D″). Scale bar: 10 µm. Merged, enlarged images of the boxed regions are shown in (B‴–D‴). (E) Ciliated cells of control RPE1 cells and the *IFT70*-KO cell lines, #70-2-11 and #70-2-12, stably expressing EGFP, EGFP-IFT70A, or EGFP-IFT70B were counted, and percentages of ciliated cells are represented as bar graphs. Values are shown as means±s.e. of three independent experiments. In each set of experiments, 62–122 cells with EGFP signals for each sample were analyzed, and the total number of cells analyzed (*n*) for each sample is shown.

As stated above, we were unable to obtain evidence for the disruption of one of the *IFT70A* alleles in both KO cell lines. Nevertheless, we performed immunoblotting analysis using a commercially available anti-IFT70 antibody to analyze the possible depletion of the IFT70 protein; the antibody was confirmed to recognize both the IFT70A and IFT70B proteins (see Fig. 3A, top panel, lanes 2 and 3). As shown in Fig. 2C, top panel, the band for IFT70 that was detected in control cells (lane 1) was not detected in the #70-2-11 and #70-2-12 cell lines (lanes 2 and 3, respectively). On the other hand, the protein level of IFT88 or IFT52, both of which directly interact with IFT70 (see below), was not substantially different between control RPE1 cells and the *IFT70*-KO cell lines (Fig. 2C, second and third panels, respectively). Thus, all of the *IFT70A* and *IFT70B* alleles are likely to be successfully disrupted in both the #70-2-11 and #70-2-12 cell lines, resulting in lack of expression of the IFT70 protein. A possible reason for not being able to detect the disruption of one of the *IFT70A* alleles by genomic PCR analysis is that primer set 1 was unable to anneal to the genomic DNA of one allele in the *IFT70*-KO lines owing to the deletion of a large region of the *IFT70A* gene. Another possibility is that the same deletion occurred in both *IFT70A* alleles. We did not pursue this point any further to confirm the disruption of the remaining *IFT70A* allele, because the ‘no-cilia’ phenotype of both the #70-2-11 and #70-2-12 cell lines were rescued by the exogenous expression of IFT70A or IFT70B (see below).

We also performed immunofluorescence analysis of control RPE1 cells and the *IFT70*-KO cell lines using the anti-IFT70 antibody. After induction of cilia formation under serum-starved conditions, IFT70 staining was found mainly at the base of cilia and faintly along cilia in control cells (Fig. 2D). In striking contrast, no cilia were formed in the #70-2-11 and #70-2-12 cell lines, analyzed by Ac-α-tubulin staining (Fig. 2E′,F′; also see Fig. 3E). Furthermore, IFT70 staining was not found in the area near the basal body (Fig. 2E,F), confirming a lack of the IFT70 protein in the #70-2-11 and #70-2-12 cell lines. The complete lack of cilia in the *IFT70*-KO cell lines was somewhat unexpected, as the zebrafish *fleer* mutant was reported to form short cilia (Pathak et al., 2007).

To exclude the possibility that the ‘no cilia’ phenotype of the #70-2-11 and #70-2-12 cell lines was a result of potential off-target effects of the CRISPR/Cas9 system, we analyzed whether the phenotype could be rescued by exogenous IFT70 expression, by establishing the #70-2-11 and #70-2-12 cell lines stably expressing EGFP-tagged IFT70A or IFT70B (Fig. 3A). As shown in Fig. 3C′ and D′, exogenous expression of either EGFP-IFT70A or EGFP-IFT70B in the #70-2-11 cell line restored ciliogenesis. Essentially the same results were obtained using the #70-2-12 cell line (Fig. 3E), demonstrating that the absence of cilia in the #70-2-11 and #70-2-12 cell lines are indeed due to lack of the IFT70 protein. These data also indicate that IFT70A and IFT70B are redundant, at least in terms of ciliogenesis.

**Fig. 4.**
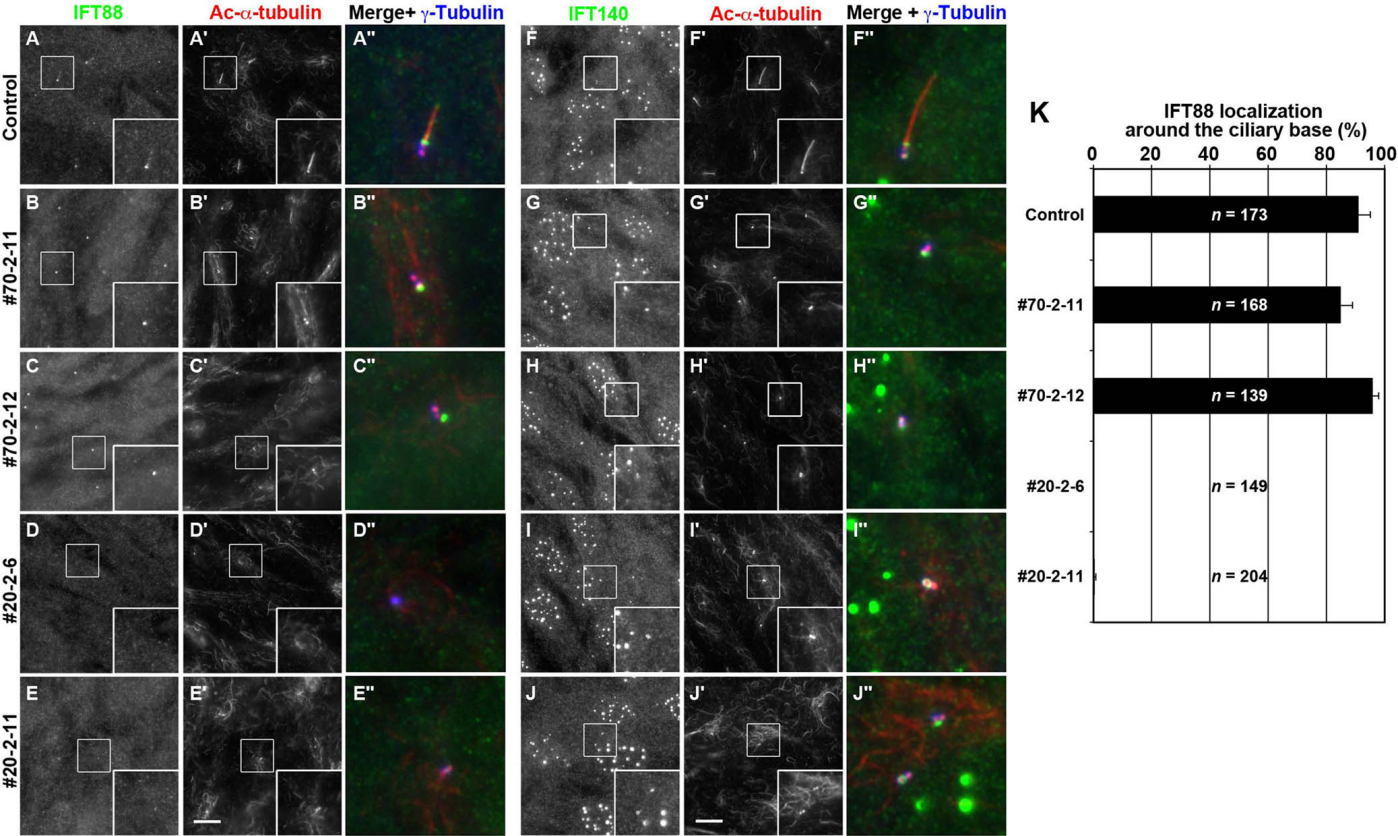
**Localization of IFT-B and IFT-A proteins in *IFT70*-KO cells.** Control RPE1 cells (A,F), the *IFT70*-KO cell lines #70-2-11 (B,G) and #70-2-12 (C,H), and the *IFT20*-KO cell lines #20-2-6 (D,I) and #20-2-11 (E,J), were serum-starved for 24 h. The cells were then triple immunostained for either IFT88 (A–E) or IFT140 (F–J), together with Ac-α-tubulin (A′–J′), and γ-tubulin (A″–J″). Scale bars: 10 µm. Merged, enlarged images of the boxed regions are shown in (A″-J″). (K) Control RPE1 cells and the *IFT70*-KO and *IFT20*-KO cell lines having IFT88 signals around the ciliary base were counted, and percentages of IFT88-positive cells are represented as bar graphs. Values are shown as means±s.e. of three independent experiments. In each set of experiments, 38–91 cells for each sample were analyzed, and the total number of cells analyzed (*n*) for each sample is shown.

We then analyzed the localization of IFT-B and IFT-A proteins in the *IFT70*-KO cell lines. In control RPE1 cells, IFT88 was localized mainly at the ciliary base and faintly throughout cilia (Fig. 4A), similarly to IFT70 (Fig. 2D). In the *IFT70*-KO cell lines, IFT88 was found at the basal body/centrosome (Fig. 4B,C) even in the absence of cilia (also see Fig. 4K). In this context, we also examined IFT88 localization in *IFT20*-KO cells, since we recently showed that *IFT20*-KO cells also lack cilia (Katoh et al., 2017). In contrast to IFT70-KO cells, however, IFT88 staining was barely detectable in the *IFT20*-KO cell lines #20-2-6 and #20-2-11 (Fig. 4D,E). These observations, taking account into the data shown in Fig. 2C, suggest that in the absence of IFT70, the rest of the IFT-B subunits can be assembled into the IFT-B complex around the basal body, but not in the absence of IFT20 (see Discussion). On the other hand, IFT140 (an IFT-A protein) was found at the base of cilia in both the *IFT70*-KO and *IFT20*-KO cell lines (Fig. 4G–J), suggesting IFT-B-independent assembly of the IFT-A complex around the basal body.

### Interactions of IFT70A and IFT70B with IFT52 and IFT88

In our previous study, we showed that human IFT70B interacts with the IFT52–IFT88 dimer in the IFT-B1 (core) subcomplex (Katoh et al., 2016), whereas Lorentzen and colleagues demonstrated a binary interaction between *Chlamydomonas* IFT52 and IFT70 (Taschner et al., 2014). To address this apparent discrepancy, we analyzed the interactions of IFT70A and IFT70B with IFT52 and IFT88 by the visible immunoprecipitation (VIP) assay, which we recently developed as a protein–protein interaction assay that can visually detect not only binary protein interactions but also one-to-many and many-to-many protein interactions (Katoh et al., 2015, 2016).

**Fig. 5.**
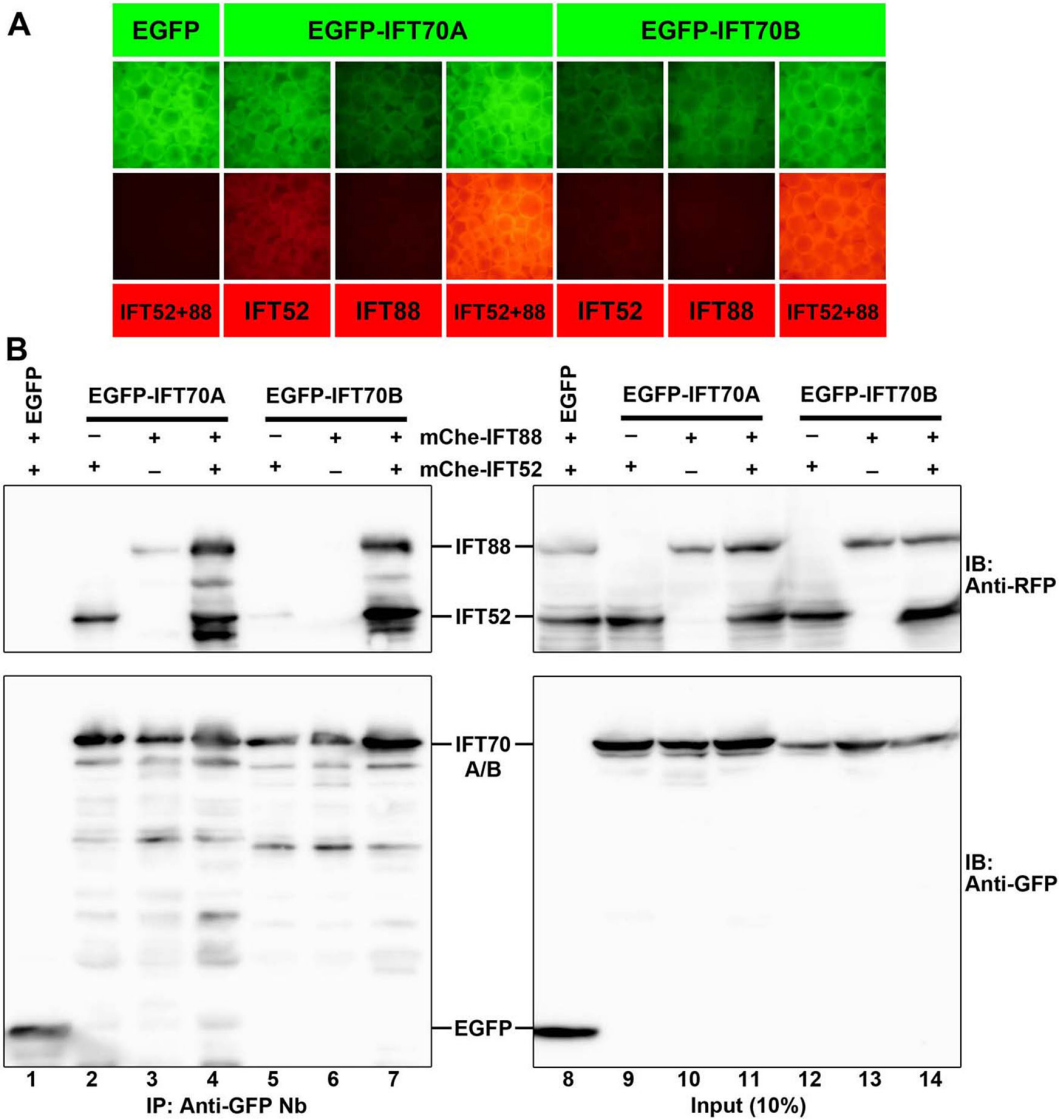
**Interaction of IFT70A and IFT70B with IFT52 and IFT88.** HEK293T cells were cotransfected with expression vectors for EGFP or EGFP-fused IFT70A or IFT70B and mChe-fused IFT52, IFT88, or IFT52+IFT88 as indicated. 24 h after transfection, lysates were prepared from the cells and precipitated with GST-tagged anti-GFP Nb pre-bound to glutathione–Sepharose beads. The beads bound to the fluorescent fusion proteins were then processed for the VIP assay (A) or immunoblotting analysis (B) using an anti-RFP antibody (upper panels) that reacts with mChe, or an anti-GFP antibody (lower panels).

Lysates were prepared from HEK293T cells cotransfected with an expression vector for EGFP-fused IFT70A or IFT70B and that for either mCherry (mChe)-fused IFT52 or IFT88 alone or in combination, and subjected to immunoprecipitation with GST-tagged anti-GFP nanobodies (Nb) prebound to glutathione-Sepharose beads. By directly observing the precipitated beads bearing fluorescent fusion proteins under a microscope, bright red signals were detected when EGFP-fused IFT70A or IFT70B was coexpressed with mChe-fused IFT52+IFT88 (Fig. 5A, columns 4 and 7). Relatively weak red signals were also detected when EGFP-IFT70A (column 2), but not EGFP-IFT70B (column 5), was coexpressed with mChe-IFT52 alone. The VIP data were confirmed by analyzing the precipitated beads by conventional immunoblotting (Fig. 5B). Intense bands for mChe-IFT52 and mChe-IFT88 were detected when they were coexpressed with EGFP-fused IFT70A or IFT70B (lanes 4 and 7), and the band for mChe-IFT52 was detected when it was coexpressed with EGFP-IFT70A (lane 2). Thus, both IFT70A and IFT70B interact robustly with the IFT52–IFT88 dimer, and IFT70A can interact weakly with IFT52 alone. These data are compatible with our previous data using human IFT70B (Katoh et al., 2016), and with the data of Lorentzen and colleagues using *Chlamydomonas* IFT70 (Taschner et al., 2014).

### The full-length IFT70A protein is required for its robust interaction with IFT52 and IFT88

**Fig. 6.**
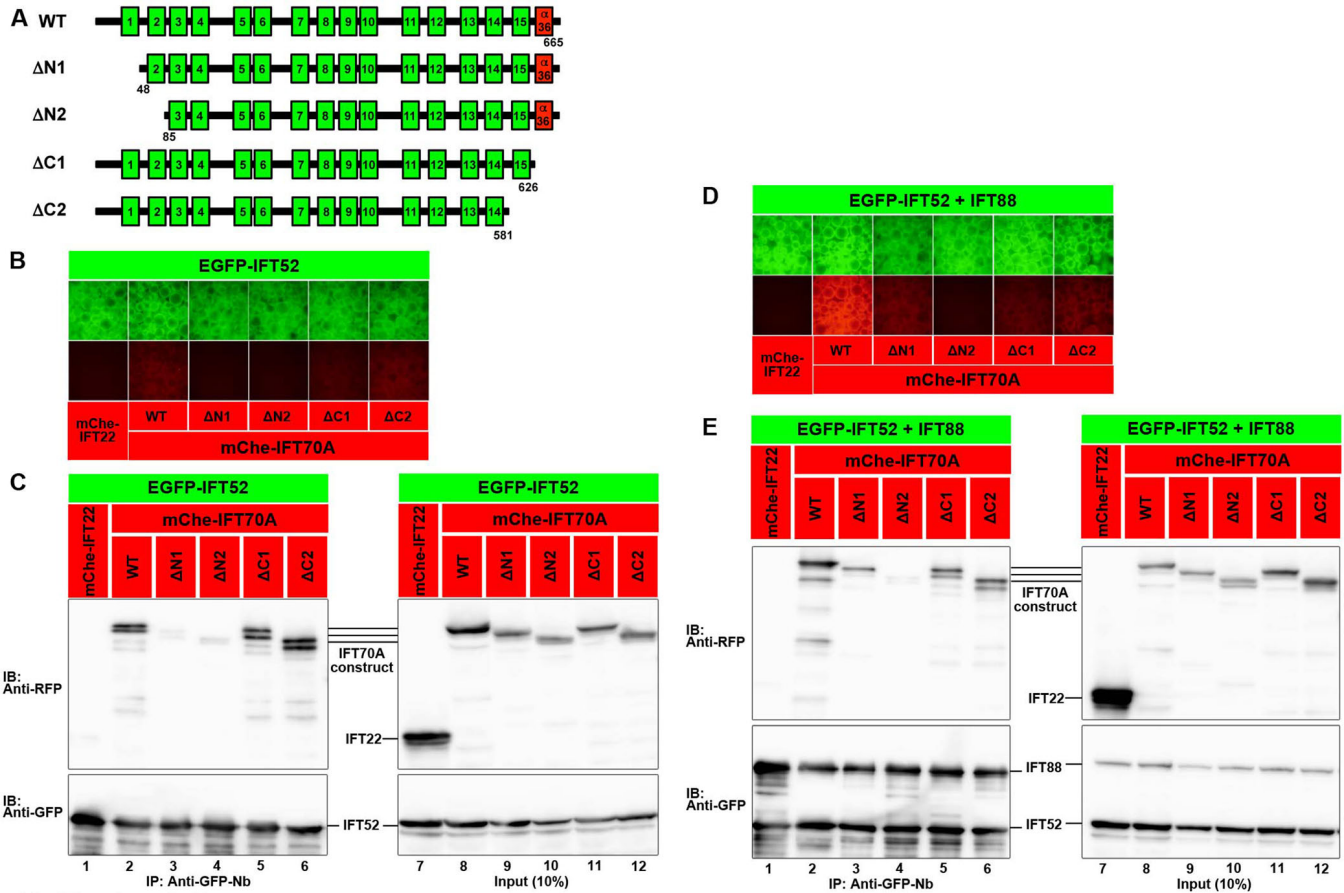
**Regions of the IFT70A protein required for its interactions with IFT52 and IFT52+IFT88.** (A) Schematic representation of the IFT70A truncation constructs. The 15 TPRs (green boxes) and α36 (red boxes) are shown. (B–E) VIP assay (B,D) and immunoblotting analysis (C,E) to determine the region of the IFT70A protein required for its interaction with IFT52 (B,C) or IFT52+IFT88 (D,E). Lysates were prepared from HEK293T cells cotransfected with expression vectors for EGFP-IFT52 (B,C) or EGFP-fused IFT52 and IFT88 (D,E), and mChe-IFT22 as a negative control or an mChe-fused IFT70A construct as indicated, and processed for the VIP assay (B,D) or immunoblotting analysis (C,E) using antibodies against RFP (upper panels) or GFP (lower panels).

X-ray crystallographic analysis of *Chlamydomonas* IFT70 in complex with IFT52 by Lorentzen and colleagues demonstrated that IFT70 adopts a superhelical structure composed of 15 tetratricopeptide repeats (TPRs) and wraps around the IFT52 middle region (Taschner et al., 2014); in the crystal structure, all 15 TPRs participate in wrapping of IFT52. We therefore constructed various truncation mutants of IFT70A and analyzed their interactions with IFT52 and IFT52+IFT88 by the VIP assay and immunoblotting analysis. When the N-terminal TPR1 was deleted (Fig. 6A, row 2), the truncated construct (ΔN1) no longer demonstrated a binary interaction with IFT52 (Fig. 6B,C, lane 3). In addition, the ΔN1 construct demonstrated an extremely reduced interaction with IFT52+IFT88 (Fig. 6D,E, lane 3). A further truncated mutant, ΔN2 (Fig. 6A, row 3), did not interact with IFT52 alone (Fig. 6B,C, lane 4) or with IFT52+IFT88 (Fig. 6D,E, lane 4). Thus, the N-terminally located TPRs are essential for the interaction of IFT70A with IFT52 and IFT52+IFT88.

TPR15 is followed by an additional C-terminal α-helix (α36) (see Fig. 6A) (Taschner et al., 2014). The X-ray crystallographic and biochemical study of *Chlamydomonas* IFT70 by Lorentzen and colleagues did not indicate the participation of α36 in the binding of IFT70 to IFT52. Consistent with this, truncation of α36 (ΔC1 construct; Fig. 6A, row 4) did not substantially affect the binding of IFT70A to IFT52 (Fig. 6B,C, lane 5). Essentially the same results were obtained using ΔC2, a further truncated mutant (Fig. 6B,C, lane 6) that lacks TPR15 in addition to α36 (Fig. 6A, row 5). The study on *Chlamydomonas* IFT70 also suggested that IFT88 is located on the C-terminal side of IFT70 (Taschner et al., 2014). In line with this, binding of IFT70A(ΔC1) and IFT70A(ΔC2) to IFT52–IFT88 was moderately compromised compared with that of IFT70A(WT) (Fig. 6D,E, compare lanes 5 and 6 with lane 2).

### The full-length IFT70A protein is required for proper function of the IFT-B complex in ciliogenesis

**Fig. 7.**
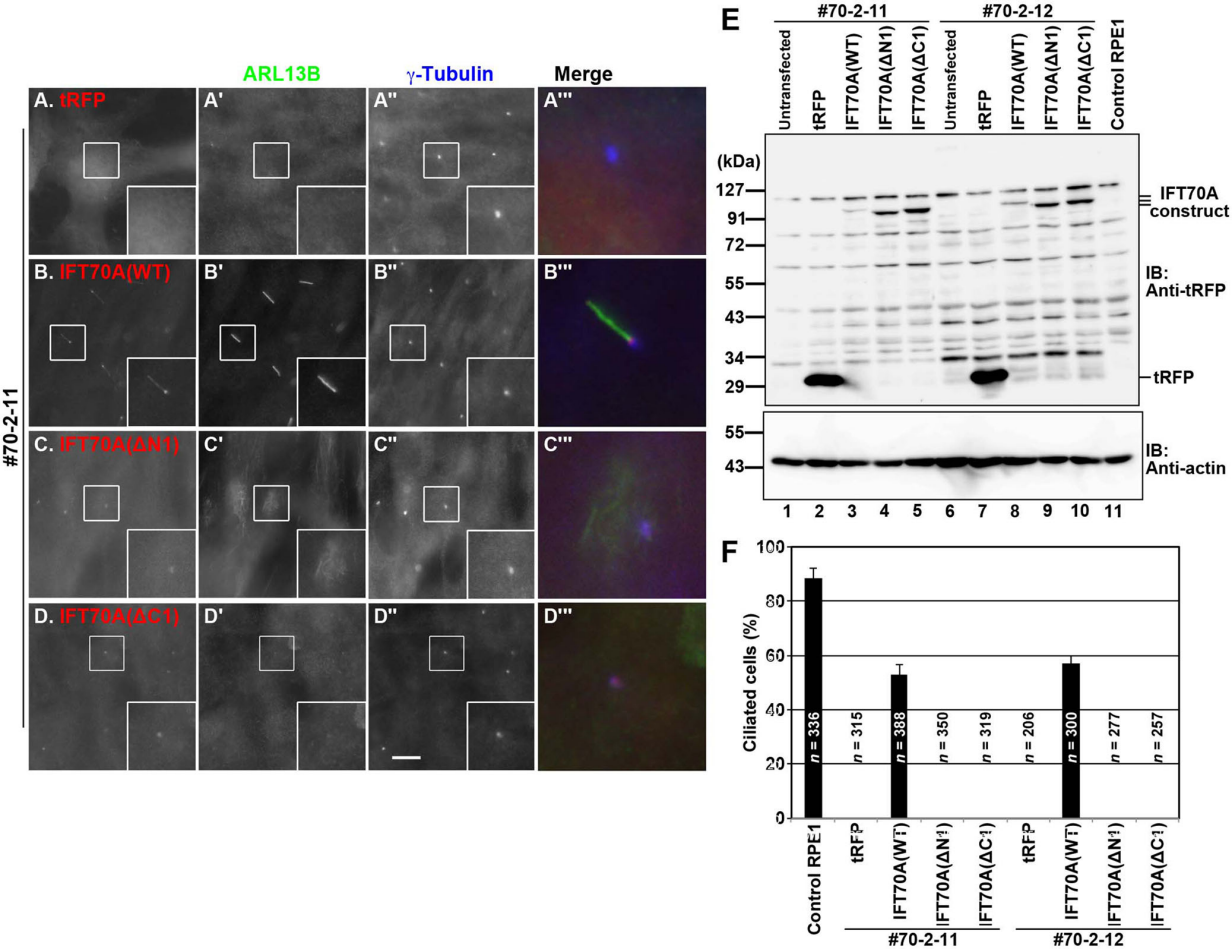
**Robust interaction of IFT70A with IFT52+IFT88 is essential for its role in ciliogenesis.** (A–D) The #70-2-11 cell line stably expressing tRFP (A), tRFP-IFT70A(WT) (B), tRFP-IFT70A(ΔN1) (C), or tRFP-IFT70A(ΔC1) (D) were serum-starved to induce ciliogenesis and double immunostained using antibodies against ARL13B (A–D′) and γ-tubulin (A′′–D′′). Scale bar: 10 µm. Merged, enlarged images of the boxed regions are shown in (A′′′–D′′′). (E) Lysates were prepared from control RPE1 cells and the *IFT70*-KO cell lines #70-2-11 and #70-2-12 stably expressing tRFP or tRFP-fused IFT70A(WT), tRFP-IFT70A(ΔN1), or tRFP-IFT70A(ΔC1), and processed for immunoblotting analysis using an anti-tRFP antibody (upper panel) or an anti-actin antibody as a control (lower panel). Note that although the commercially available anti-tRFP antibody gave rise to a number of nonspecific bands, the bands for exogenously expressed tRFP-fused IFT70A constructs were clearly detected. (F) Ciliated cells of control RPE1 cells and the *IFT70*-KO cell lines #70-2-11 and #70-2-12 stably expressing tRFP or tRFP-fused IFT70A(WT), tRFP-IFT70A(ΔN1), or tRFP-IFT70A(ΔC1) were counted, and percentages of ciliated cells are represented as a bar graph. Data are shown as means±s.e. of three independent experiments. In each experiment, 56–146 cells with tRFP signals for each sample were analyzed, and the total number of cells analyzed (*n*) for each sample is shown.

We then analyzed whether the IFT70A truncation mutants confirmed above can rescue the ciliogenesis defect of *IFT70*-KO cells. In contrast to tRFP-IFT70A(WT) (Fig. 7B), the exogenous expression of neither tRFP-IFT70A(ΔN1) (Fig. 7C–C′′′) nor tRFP-IFT70A(ΔC1) (Fig. 7D–D′′′) was able to restore ciliogenesis in the *IFT70*-KO cell line #70-2-11 (also see Fig. 7F); expression of the IFT70A constructs in *IFT70*-KO cells was confirmed by immunoblotting analysis (Fig. 7E). Essentially the same results were obtained using the other *IFT70*-KO cell line (#70-2-12) (Fig. 7F). Taking into account the data of the binding of IFT70A constructs to IFT52 and the IFT52–IFT88 dimer (Fig. 6), it is thus likely that a robust interaction of IFT70 with the IFT52–IFT88 dimer is essential for the function of IFT-B in ciliogenesis, although we cannot completely exclude the possibility that IFT70A(ΔN1) and/or IFT70A(ΔC1) dominant negatively affected ciliogenesis via their weak interaction with IFT52–IFT88 (Fig. 6D,E).

## DISCUSSION

In this study, we established KO cell lines of IFT22 and IFT70, both of which are located at the periphery of the IFT-B1 subcomplex. *IFT22*-KO cells showed no apparent defects in ciliogenesis or trafficking of the ciliary proteins that were analyzed. These results were somewhat contrary to our expectation that the small GTPase IFT22/RABL5 might regulate ciliary functions, because IFT27/RABL4 and RABL2, which interact with the IFT74–IFT81 heterodimer similarly to IFT22/RABL5 (Kanie et al., 2017; Katoh et al., 2016; Nishijima et al., 2017), participate in ciliary protein trafficking (Eguether et al., 2014) and ciliogenesis (Kanie et al., 2017; Nishijima et al., 2017). Furthermore, knockdown of IFT22 in *Trypanosoma* and in *Chlamydomonas* was reported to result in short flagella (Adhiambo et al., 2009; Silva et al., 2012), although disruption of the *IFT22* gene in *C. elegans* did not have any effects on ciliary assembly or protein trafficking (Schafer et al., 2006).

*Drosophila melanogaster* lacks IFT22, IFT25, and IFT27 among the IFT-B subunits (Shida et al., 2010). As cells in *Drosophila* do not generally have cilia, except for several specialized cells, these three IFT-B subunits might play auxiliary roles in ciliary protein trafficking. Indeed, the IFT27 in complex with IFT25 was proposed to regulate retrograde protein trafficking by connecting the IFT-B complex to the BBSome, although ciliogenesis was not apparently affected in the absence of IFT25 or IFT27 (Eguether et al., 2014; Huet et al., 2014; Keady et al., 2012; Liew et al., 2014). It is therefore possible that IFT22 has an unidentified role in ciliary protein trafficking.

In marked contrast to *IFT22*-KO cells, cells simultaneously knocked out of the *IFT70A* and *IFT70B* genes could not form cilia. However, unlike *IFT20*-KO cells, in which the IFT-B complex cannot be assembled, the remaining IFT-B subunits appeared to assemble to form a complex at the ciliary base in *IFT70*-KO cells. The ciliogenesis defect of *IFT70*-KO cells was rescued by the exogenous expression of IFT70A or IFT70B, indicating redundant roles of IFT70A and IFT70B.

Analyses of the exogenous expression of truncation mutants of IFT70A in *IFT70*-KO cells suggest that the incorporation of IFT70 into the IFT-B complex via its robust interaction with the IFT52–IFT88 dimer is essential for ciliogenesis. Ciliogenesis defects are likely caused by the lack of trafficking of tubulins, which are building blocks of axonemal microtubules. In the IFT-B complex, IFT74 and IFT81, which form a tight complex via their coiled-coil regions, bind to the αβ-tubulin dimer via their N-terminal regions (Bhogaraju et al., 2013; Kubo et al., 2016). It was recently reported that IFT54 can also bind to tubulins (Taschner et al., 2016). In our architectural model of the IFT-B complex (Katoh et al., 2016), however, IFT70 is not directly linked to or in close proximity to IFT74, IFT81, or IFT54 (see Fig. 1A). As proteins and protein complexes are three-dimensional entities, IFT70 may be spatially close to any of these IFT-B subunits, to be able to regulate the binding of tubulin to, or tubulin trafficking by, the IFT-B complex.

Another possibility is that in the absence of IFT70, the IFT-B complex cannot enter cilia across the transition zone. Our analyses using IFT70A truncation mutants showed that the entire length of the IFT70 protein is required for its robust interaction with IFT52 and IFT52–IFT88. Taking into account the crystal structure of *Chlamydomonas* IFT70, in which almost the entire region of the IFT70 protein participates in wrapping around the IFT52 middle region (Taschner et al., 2014), IFT70 does not appear to interact directly with IFT-B subunits or an unidentified protein other than IFT52 and IFT88. In this context, it is interesting to note the work of Zhao and Malicki (Zhao and Malicki, 2011), in which the transition zone protein B9D2 was shown to interact with IFT70B by the yeast two-hybrid assay. However, our preliminary analysis using the VIP assay has so far been unable to show a specific interaction between B9D2 and either IFT70A or IFT70B. It is therefore possible that binding of IFT70 to IFT52–IFT88 induces a subtle conformational change in the IFT-B complex, which enables its entry into cilia.

## MATERIALS AND METHODS

### Plasmids, antibodies, and reagents

Expression vectors for human IFT70A (reference sequence: NM_152275.3) and its deletion constructs, and for IFT70B (reference sequence: NM_152517.2) used in this study are listed in Table S1; several of them were constructed in our previous study (Katoh et al., 2016). The antibodies used in this study are listed in Table S2. GST-tagged anti-GFP Nbs prebound to glutathione–Sepharose 4B beads were prepared as described previously (Katoh et al., 2015).

### VIP assay and immunoblotting analysis

The VIP assay and subsequent immunoblotting analysis were performed by a previously described method (Katoh et al., 2015, 2016) with minor modifications (Nishijima et al., 2017). Briefly, approximately 1.6×10^6^ HEK293T cells in six-well plates were transfected with EGFP and mChe fusion constructs using Polyethylenimine Max (20 µg, Polysciences, Warrington, USA), and cultured for 24 h. The transfected cells were then lysed for 20 min on ice in 250 µl of HMDEKN cell lysis buffer [10 mM HEPES (pH 7.4), 5 mM MgSO_4_, 1 mM DTT, 0.5 mM EDTA, 25 mM KCl, and 0.05% NP-40] containing EDTA-free protease inhibitor cocktail (Nacalai Tesque, Kyoto, Japan), and centrifuged at 16,100×***g*** for 15 min at 4°C in a microcentrifuge. The supernatants (200 µl) were incubated with 5 µl of GST–anti-GFP Nb beads for 1 h at 4°C. After washing three times with 180 µl of the cell lysis buffer, the precipitated beads were observed using an all-in-one-type fluorescence microscope (BZ-8000, Keyence, Osaka, Japan) using a 20×/0.75 objective lens under constant conditions (sensitivity ISO 400, exposure 1/30 s for green fluorescence; and sensitivity ISO 800, exposure 1/10 s for red fluorescence).

### Immunofluorescence analysis

hTERT-RPE1 cells (American Type Culture Collection, CRL-4000, Manassas, USA) were cultured in DMEM/F-12 (Nacalai Tesque) supplemented with 10% fetal bovine serum (FBS) and 0.348% sodium bicarbonate. To induce ciliogenesis, cells were grown to 100% confluence on coverslips, and starved for 24 h in Opti-MEM (Invitrogen) containing 0.2% bovine serum albumin. Subsequent immunofluorescence analysis was performed as described previously (Hirano et al., 2017; Takahashi et al., 2012). The cells were fixed and permeabilized with 3% paraformaldehyde at 37°C for 5 min and subsequently in methanol at −20°C for 5 min, and washed three times with phosphate-buffered saline. For detection of endogenous IFT140, cells were fixed and permeabilized with methanol at −20°C for 5 min, and washed three times with phosphate-buffered saline. The fixed/permeabilized cells were blocked with 10% FBS and stained with antibodies diluted with 5% FBS. The stained cells were observed using an Axiovert 200 M microscope (Carl Zeiss, Jena, Germany). Statistical analyses were performed using JMP Pro 12 software (SAS Institute, Tokyo, Japan).

### Establishment of KO cell lines using the CRISPR/Cas9 system

The strategy of CRISPR/Cas9-mediated gene disruption of hTERT-RPE1 cells using homology-independent DNA repair was previously reported in detail (Katoh et al., 2017); also see (Funabashi et al., 2017; Hirano et al., 2017; Nishijima et al., 2017; Nozaki et al., 2018, 2017; Takahara et al., 2018). Single guide RNA (sgRNA) sequences targeting the *IFT22* gene or those targeting both the *IFT70A* and *IFT70B* genes (see Table S3) were designed using CRISPR design (Hsu et al., 2013). We applied the version 2 system of a combination of the donor knock-in vector and the all-in-one sgRNA expression vector pDonor-tBFP-NLS-Neo(Universal) (Addgene #80767, Cambridge, USA) and peSpCas9(1.1)-2×sgRNA (Addgene #80768), respectively (Katoh et al., 2017). hTERT-RPE1 cells were grown to approximately 3.0×10^5^ cells in a 12-well plate, and transfected with 1 µg of the sgRNA vector and 0.25 µg of the donor vector using X-tremeGENE9 reagent (Roche Life Science, Pleasanton, USA). After selection in the presence of G418 (600 µg/ml), colonies of cells with nuclear tBFP signals were isolated under a microscope. Genomic DNA was extracted from the isolated cells and subjected to PCR using the primer sets listed in Table S3. Details of the characterization of the *IFT22*-KO and *IFT70*-KO cell lines are shown in Fig. S1, and Fig. 2 and Fig. S2, respectively.

### Preparation of cells stably expressing EGFP-IFT70

Lentiviral vectors for IFT70A and IFT70B were constructed as described previously (Takahashi et al., 2012). Briefly, pRRLsinPPT-EGFP/tRFP-IFT70A(WT), pRRLsinPPT-tRFP-IFT70A(ΔN1), pRRLsinPPT-tRFP-IFT70A(ΔC1), or pRRLsinPPT-EGFP/tRFP-IFT70B(WT) was transfected into HEK293T cells together with packaging plasmids [pRSV-REV, pMD2.g, and pMDL/pRRE; kindly provided by Peter McPherson, McGill University (Thomas et al., 2009)]. The culture medium was replaced 8 h after transfection, and collected between 24–48 h after transfection. The medium containing lentiviral particles was passed through a 0.45-µm filter and centrifuged at 32,000×***g*** at 4°C for 4 h. The precipitated viral particles were resuspended in Opti-MEM (Invitrogen) and stored at −80°C until use.

## Supplementary Material

Supplementary information
