# Supplementary material for: Robust interaction of IFT70 with IFT52–IFT88 in the IFT-B complex is required for ciliogenesis
Source: Biol Open. 2018 Apr 13;7(5):bio033241. doi: 10.1242/bio.033241 (PMC5992529; doi:10.1242/bio.033241)
Supplement: Supplementary information [file biolopen-7-033241-s1.pdf]

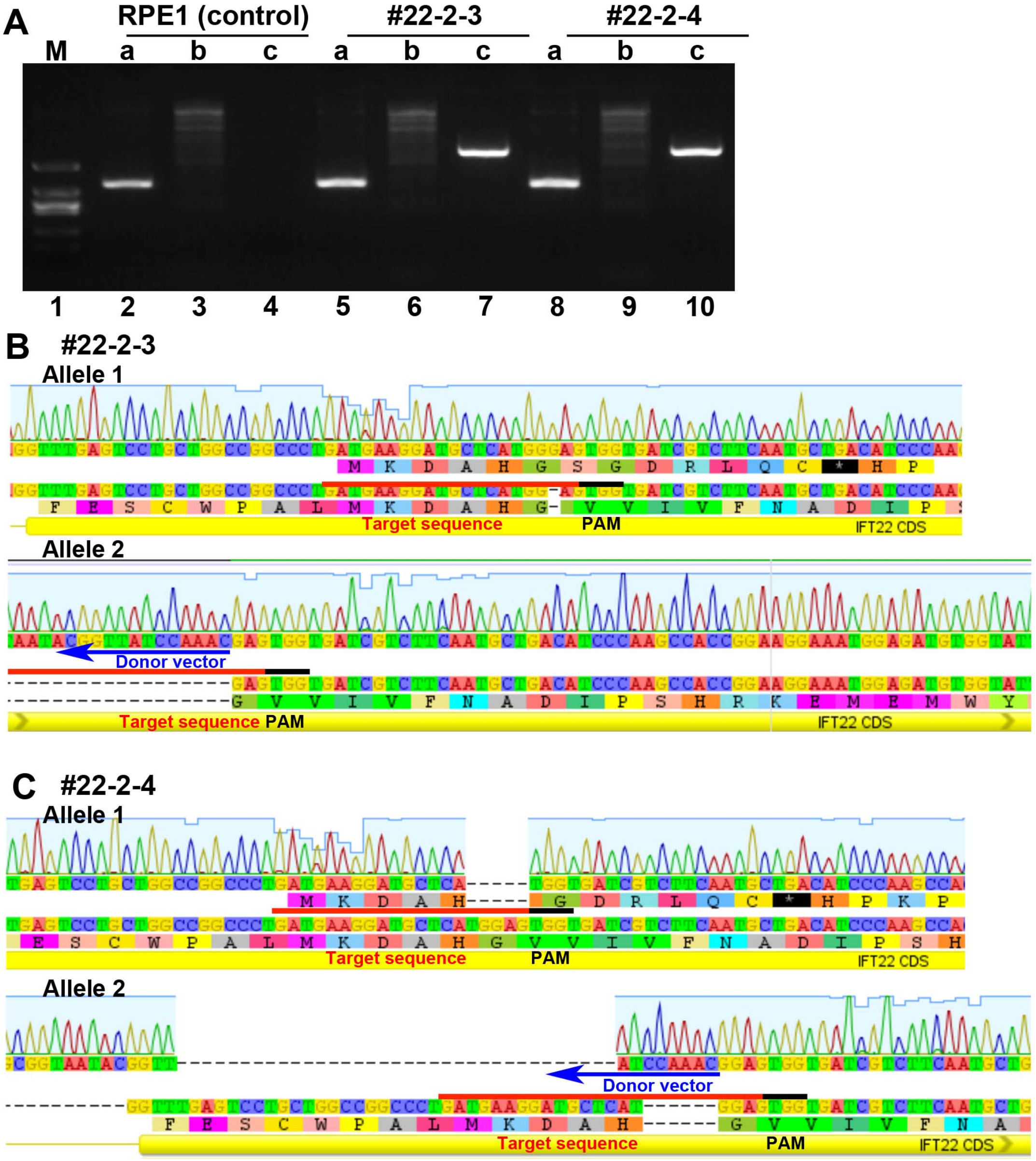

Fig. S1. Genomic PCR and sequencing to confirm donor vector integration in selected *IFT22*-KO cell lines

(A) Genomic DNA was extracted from control RPE1 cells (lanes 2–4), and from the *IFT22*-KO cell lines (#22-2-3, lanes 5–7; and #22-2-4, lanes 8–10) established using a donor knock-in vector. The DNA was subjected to PCR using primer pair a (primers G + H; lanes 2, 5, and 8), pair b (primers C + G; lanes 3, 6, and 9), and pair c (primers C + H; lanes 4, 7, and 10) (see Table S3). (B and C) Alignments of allele sequences of cell lines #22-2-3 (B) and #22-2-4 (C) determined by direct sequencing of the genomic PCR products with the reference sequence encompassing the IFT22 coding sequence. Red and black lines indicate the target sequences and protospacer adjacent motif (PAM) sequence, respectively. Blue arrows indicate the direction of vector integration. The #22-2-3 cell line has a one-nucleotide insertion in one IFT22 allele and a reverse integration of the donor knock-in vector in the other allele (B); and the #22-2-4 cell line has a five-nucleotide deletion in one IFT22 allele and a reverse integration of the donor knock-in vector in the other allele (C).

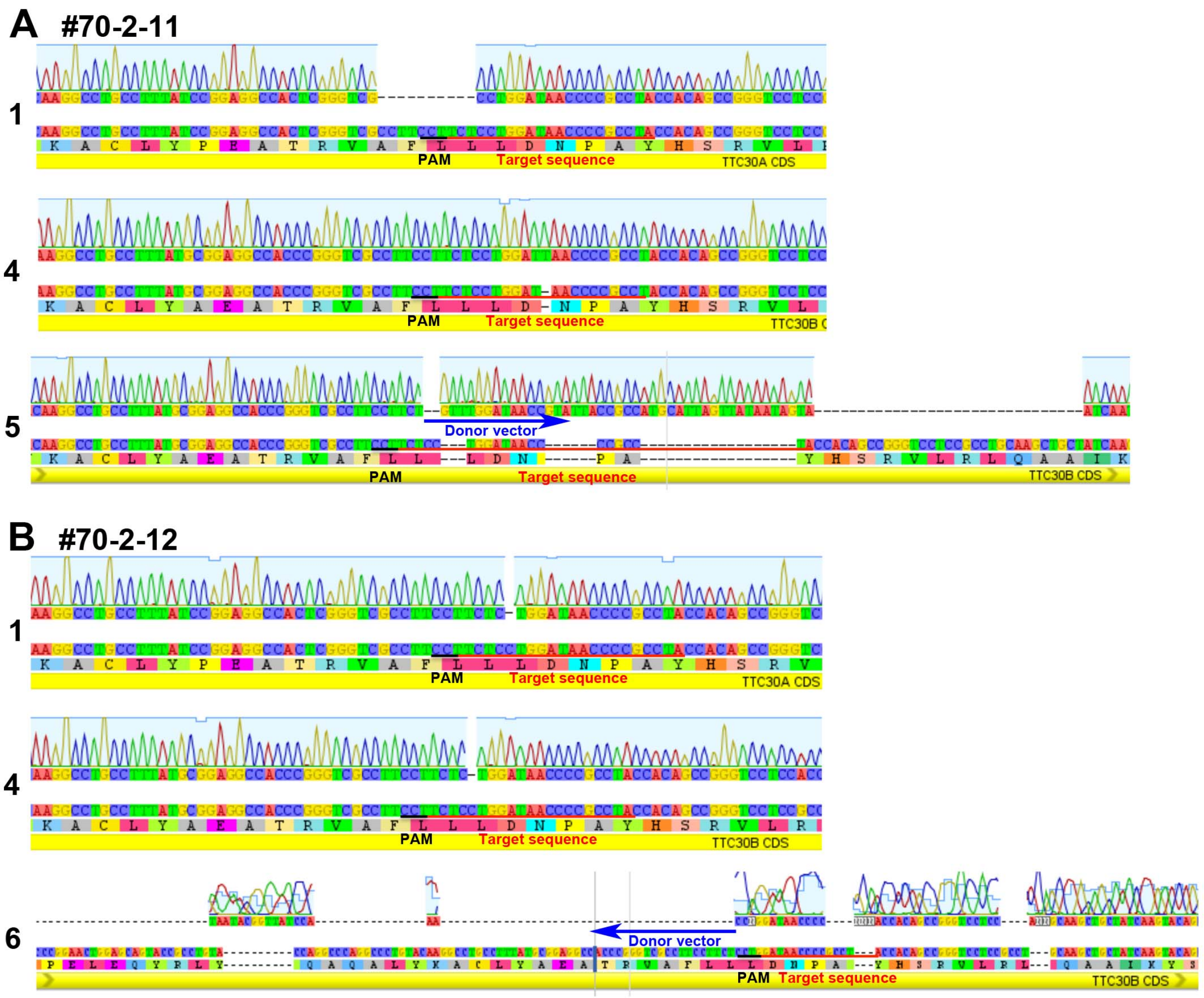

Fig. S2. Alignments of allele sequences of the *IFT70*-KO cell lines

(A) The PCR products of the #70-2-11 genomic DNA shown in Fig. 2B, middle panel, lanes 1, 4, and 5, and those of the #70-2-12 genomic DNA shown in Fig. 2B, bottom panel, lanes 1, 4, and 6, were subjected to direct sequence analysis with the reference sequence encompassing the IFT70 coding sequence. Red and black lines indicate the target sequence and PAM sequence, respectively. Blue arrows indicate the direction of vector integration. The #70-2-11 cell line has a 10-nucleotide deletion in one of the *IFT70A* alleles, a one-nucleotide insertion in one of the *IFT70B* alleles, and a forward integration of the donor vector in the other *IFT70B* allele; and the #70-2-12 cell line has a one-nucleotide deletion in one of the *IFT70A* alleles, a one-nucleotide deletion in one of the *IFT70B* alleles, and a reverse integration of the vector in the other *IFT70B* allele.

Table S1. Plasmid vectors used in this study

No	Vector	Insert	Reference
1	pCAG2-EGFP-C	Human IFT70A	This study
2	pCAG2-mCherry-C	Human IFT70A	This study
3	pCAG2-mCherry-C	Human IFT70A(ΔN1:48-665)	This study
4	pCAG2-mCherry-C	Human IFT70A(ΔN2:85-665)	This study
5	pCAG2-mCherry-C	Human IFT70A(ΔC1:1-626)	This study
6	pCAG2-mCherry-C	Human IFT70A(ΔC2:1-581)	This study
7	pCAG2-EGFP-C	Human IFT70B	Katoh et al. (2016)
8	pRRLsinPPT-EGFP-C	Human IFT70A	This study
9	pRRLsinPPT-EGFP-C	Human IFT70B	This study
10	pRRLsinPPT-tRFP-T-C	Human IFT70A	This study
11	pRRLsinPPT-tRFP-T-C	Human IFT70A(ΔN1:48-665)	This study
12	pRRLsinPPT-tRFP-T-C	Human IFT70A(ΔC1:1-626)	This study
13	pCAG-EGFP-C	Human IFT52	Katoh et al. (2016)
14	pCAG-EGFP-C	Human IFT88	Katoh et al. (2016)
15	pCAG-mCherry-C	Human IFT52	Katoh et al. (2016)
16	pCAG-mCherry-C	Human IFT88	Katoh et al. (2016)
17	pmCherry-C	Human IFT22	This study

Biology Open • Supplementary information

Table S2. Antibodies used in this study

Antibody	Manufacturer	Clone or catalog number	Dilution (purpose)
Monoclonal mouse anti-Ac-α-tubulin	Sigma-Aldrich	6-11B-1	1:500 (IF)
Monoclonal mouse anti-γ-tubulin	Sigma-Aldrich	GTU88	1:1,000 (IF)
Polyclonal rabbit anti-IFT139	Sigma-Aldrich	HPA035495	1:1000 (IB)
Polyclonal rabbit anti-ARL13B	Proteintech	17711-1-AP	1:500 (IF)
Polyclonal rabbit anti-IFT88	Proteintech	13967-1-AP	1:200 (IF); 1:1,000 (IB)
Polyclonal rabbit anti-IFT140	Proteintech	17460-1-AP	1:100 (IF)
Polyclonal rabbit anti-IFT70	Proteintech	25352-1-AP	1:100 (IF); 1:500 (IB)
Polyclonal rabbit anti-IFT52	Proteintech	17534-1-AP	1:1,000 (IB)
Polyclonal rabbit anti-ARL13B	Proteintech	17711-1-AP	1:1,000 (IF)
Polyclonal rabbit anti-GPR161	Proteintech	13398-1-AP	1:200 (IF)
Monoclonal mouse anti-GFP	BD Biosciences	JL-8	1:1,000 (IB)
Polyclonal rabbit anti-RFP	MBL Life Science	PM005	1:1,000 (IB)
Polyclonal rabbit anti-tRFP	Evrogen	AB233	1:1,000 (IB)
Monoclonal mouse anti-β-tubulin	EMD Millipore	KMX-1	1:2,000 (IB)
Monoclonal mouse anti-actin	EMD Millipore	C4	1:2,000 (IB)
AlexaFluor-conjugated secondary	Molecular Probes	A11034, A21240, A21147	1:1,000 (IF)
Peroxidase-conjugated secondary	Jackson ImmunoResearch	115-035-166, 111-035-144	1:3,000 (IB)

IF, immunofluorescence; IB, immunoblotting.

Table S3. Oligo DNAs used in this study

No.	Name	Sequence
1	pTagBFP-N-RV2 (primer C)	5'-CGTAGAGGAAGCTAGTAGCCAGG-3'
2	IFT70A-genome-FW (primer A)	5'- CGTGGCAAAGTAACCCGTCG -3'
3	IFT70A-genome-RV (primer B)	5'- CTTGGTGAGTCTCCGAAGCTG -3'
4	IFT70B-genome-FW (primer D)	5'- TGCGACAAGGAAACCGGCAG -3'
5	IFT70B-genome-RV (primer E)	5'- TATGGTAAGTTTCCGGAGGAC -3'
6	Donor-vector-Seq-FW (primer F)	5'- CACCTCTGACTTGAGCGTCG-3'
7	IFT70-gRNA#2-S	5'- CACCGAGGCGGGGTTATCCAGGAGA-3'
8	IFT70-gRNA#2-AS	5'- AAACTCTCCTGGATAACCCCGCCTC-3'
9	IFT22-genome-FW (primer G)	5'- ATAGGTGCCCACAACCACAC-3'
10	IFT22-genome-RV (primer H)	5'- GCCTAGGGTACAGTAGGTGC-3'
11	IFT22-gRNA#2-S	5'- CACCGATGAAGGATGCTCATGGAG-3'
12	IFT22-gRNA#2-AS	5'- AAACCTCCATGAGCATCCTTCATC-3'

Biology Open • Supplementary information
